# Editorial: Mechanisms of Inflammation and Fibrosis Interplays in the Digestive Diseases

**DOI:** 10.3389/fphys.2022.906742

**Published:** 2022-04-14

**Authors:** Atsushi Masamune, Shin Hamada

**Affiliations:** Division of Gastroenterology, Tohoku University Graduate School of Medicine, Sendai, Japan

**Keywords:** all-trans retinoic acid, anti-stromal therapy, meflin, pancreatic cancer, pancreatic fibrosis, pancreatic stellate cells, vitamin D

Fibrosis denotes excessive scarring and is seen in a wide variety of benign and malignant diseases in the digestive systems. Fibrosis develops as the consequences of inflammation, which involves a variety of components such as immune cells, fibroblasts, endothelial cells, cancer cells, mediators, extracellular matrix (ECM) components and pathogens (Erkan et al., 2012; Iwata et al., 2021; Wang et al., 2022). The dynamic interaction of these participants determines the nature of fibrosis in quantity and quality, and the fate of fibrosis. Fibrosis is now regarded as a highly dynamic process which might be reversible and serve as promising therapeutic targets. In this Research Topic, articles dealing with the roles of the inflammation and fibrosis in a variety of digestive diseases including inflammatory bowel diseases, liver fibrosis, pancreatitis, and pancreatic cancer have been published.

Pancreatic fibrosis is a characteristic pathological feature of pancreatic ducal adenocarcinoma (PDAC) (Masamune and Shimosegawa, 2013). It has been controversial whether stroma drives pancreatic cancer progression or acts as a defense against pancreatic cancer. Stroma might stimulate aggressive behaviors of pancreatic cancer cells and help them escape from host immune-surveillance. On the other hand, stroma might provide a barrier limiting dissemination and metastasis of pancreatic cancer cells. Another aspect of stroma is low blood perfusion. Dense stroma impairs drug delivery by several mechanisms such as providing physical barrier, high interstitial pressure, and less leaky phenotype of pericytes (Erkan, 2013). Fibrosis also contributes to the formation of immunosuppressive microenvironment by altering cellular interactions and differentiation of stromal cells (Hamada et al., 2022). Based on these findings, the concept of “anti-fibrosis therapy” has been proposed for the treatment of PDAC. By targeting stroma, we might expect inhibition of stroma-stimulated aggressive behaviors of pancreatic cancer cells, more efficient delivery of anti-cancer drugs within the tumor, activation of immune systems targeting cancer cells. However, metastasis and dissemination might be also increased by destroying physical barriers and increased angiogenesis. In addition, non-specific inhibition of stromal cells might abolish their host-defensive roles.

The break-through in this research field is the identification and characterization of pancreatic stellate cells (PSCs) back to 1998 (Apte et al., 1998; Bachem et al., 1998). It has been established that PSCs play a pivotal role in the development of pancreatic fibrosis in PDAC (Erkan, 2013). Previous studies have shown the cancer-promoting effects of PSCs; PSCs facilitate local tumor growth and distant metastasis, play a role in the resistance to conventional therapies, induce epithelial-mesenchymal transition and stem-cell like phenotypes, cause impaired immune responses (Hamada et al., 2022). Based on the studies using cultured PSCs, anti-fibrosis therapies appeared to be a promising approach for the treatment of PDAC. For example, vitamin D analog enhanced the pancreatic cancer therapy. Vitamin D analog (Calcipotriol) increased the decreased the tumor volume, increased the vascularity and intratumoral gemcitabine concentration, leading to improved overall survival of the KPC mice compared with gemcitabine alone (Sherman et al., 2014). However, depletion of stromal by deletion of Shh, IPI-926, or targeting *α*-smooth muscle actin (SMA)-positive myofibroblasts, unexpectedly, caused the undifferentiated cancer phenotype, increased distant metastasis and cachexia, resulting in decreased overall survival (Rhim et al., 2014). Therefore, there existed discrepancies between the results obtained using cultured PSCs and those obtained by Hedgehog inhibitors or targeting *α*-SMA in genetically engineered mouse models. It is also noteworthy that the several clinical trials assessing the effectiveness of PSC-targeting therapies failed to show effectiveness (Vaish et al., 2021). The clinical trial evaluating efficacy of IPI-926 with gemcitabine has been halted due to unexpected shortening of survival in patients with advanced pancreatic cancer, who received combination therapy. Another agent Vismodegib, an inhibitor of hedgehog inhibitor, also failed to show clinical benefits. Simutuzumab, an inhibitor of ECM remodeling enzyme, did not improve clinical outcomes. Even though the retrospective study identified longer progression-free survival of patients with pancreatic cancer receiving angiotensin I-converting enzyme inhibitors or angiotensin II type-1 receptor blockers (ARBs), the multicenter study that assessed the efficacy of candesartan, an ARB, with gemcitabine failed to show effectiveness for pancreatic cancer (Nakai et al., 2013). Stromal biology might be context-dependent and more complicated than previously recognized.

Following studies further proved this concept. The glycosylphosphatidylinositol GPI-anchored protein, meflin, is a marker of mesenchymal stem cells (MSCs). Deletion of meflin in the KPC mice resulted in the promotion of pancreatic cancer. Meflin-expressing *α*-SMA positive cells suppressed tumorigenicity and poor differentiation of pancreatic cancer cells in the subcutaneous implantation model, suggesting a certain cancer-inhibiting population in PSCs. Reduced stromal meflin expression correlated with poor prognosis of patients with PDAC (Mizutani et al., 2019). Similarly, another cancer-promoting switch of PSCs also has been identified. The Rho effector protein kinase N2 (PKN2) is essential for the myofibroblast phenotypes such as *α*-SMA expression, proliferation and contractility. Loss of PKN2 in PSCs promoted pancreatic cancer cell growth and invasion in the *in vitro* co-culture model and *in vivo* orthotopic implantation model. PKN2 knockout in PSCs affected matrisome, the component list of extracellular matrix proteins, toward pro-metastatic signature (Murray et al., 2022). In contrast, cancer-promoting subpopulations of PSCs also have been identified. Interestingly, these PSCs are further divided into myofibroblastic and inflammatory subtypes, which produce collagen fibers and inflammatory cytokines, respectively (Zhang et al., 2021). These studies highlighted the heterogeneity of PSCs, cancer-promoting and cancer-inhibiting roles, which should be taken into consideration for therapeutic intervention.

Origin of these cancer-promoting stromal cells has been extensively studied. In addition to the activation of resident fibroblast, PSCs and MSCs, cells from non-fibroblastic lineage such as epithelial cells, endothelial cells, adipocytes, pericytes and smooth muscle cells undergo transdifferentiation to support pancreatic cancer progression (Manoukian et al., 2021). Among these wide variety of cellular origins, PSCs remain numerically minor population of cancer-associated fibroblasts, whereas play nonredundant role such as specific ECM production (Helms et al., 2022). The cancer-promoting and cancer-inhibiting stromal cells’ phenotypes also can be converted with each other, according to microenvironment (Sunami et al., 2020). Numerous markers of these complex subtypes have been identified such as cell-surface molecules, receptors and cytokines.

Based on these lines of evidence, several clinical studies are ongoing. Quiescent PSCs store vitamin A-containing lipid droplet. The vitamin A-metabolite, all-trans retinoic acid (ATRA) has been shown to suppress fibrosis formation by PSCs and PDAC progression. Administration of ATRA with gemcitabine to the KPC mice efficiently reduced cancer cell proliferation and invasion, as well as the PSC-derived ECM deposition within tumor (Carapuca et al., 2016). ATRA treatment caused remodeling of pancreatic cancer microenvironment, such as increased vascularity and improvement of hypoxia. The phase I clinical trial using ATRA has been conducted by enrolling unresectable PDAC patients. Administration of gemcitabine-nab-paclitaxel along with ATRA was safe and tolerable, and increased true diffusion values by diffusion-weighted magnetic resonance imaging, suggesting altered stromal structure (Kocher et al., 2020). The number of *α*-SMA positive cells within surgically resected pancreatic cancer specimens correlated with lower plasma levels of vitamin D (Mukai et al., 2018). Clinical trial targeting vitamin D is also under its way. The phase III trial administrating high-dose oral vitamin D3 prior to surgery is ongoing, evaluating postoperative mortality (Hosein et al., 2020). Further studies are warranted to identify adequate intervention to normalize pancreatic cancer stroma. Specific cancer-promoting functions of cancer stroma also need to be clarified, for novel therapeutic targets.
